# Authors’ Response to Peer Reviews of “The Role of Animal-Assisted Therapy in Enhancing Patients’ Well-Being: Systematic Study of the Qualitative and Quantitative Evidence”

**DOI:** 10.2196/55899

**Published:** 2024-03-18

**Authors:** Ramendra Pati Pandey, Himanshu, Gunjan, Riya Mukherjee, Chung-Ming Chang

**Affiliations:** 1 School of Health Sciences & Technology, University of Petroleum and Energy Studies, Dehradun, Uttarakhand, India; 2 Graduate Institute of Biomedical Sciences, Chang Gung University, Taoyuan, Taiwan; 3 Master & PhD Program in Biotechnology Industry, Chang Gung University, Taoyuan, Taiwan; 4 Department of Medical Biotechnology and Laboratory Science, Chang Gung University, Taoyuan, Taiwan

**Keywords:** animal-assisted therapy, pet therapy, outcome assessment, policies, systematic study

*This is the authors’ response to peer-review reports for the paper “The Role of Animal-Assisted Therapy in Enhancing Patients’ Well-Being: Systematic Study of the Qualitative and Quantitative Evidence.”*

## Round 1 Review

### Anonymous [1]

#### General Comments

*I enjoyed reading this paper [* *2* *]. In general, this is a well-written paper. There are some areas that could be clarified or expanded to improve the strength of the article. Due to the justification, the spacing of punctuation marks appears incorrect, and there are rare instances of double punctuation (periods). The use of American Psychological Association abbreviations at first use was not followed. At times, after providing an abbreviation, the full name is spelled out (eg, “AAT,” “PTSD”).*

Response: We would like to express our heartfelt appreciation for your valuable and pertinent feedback. Your insights have greatly contributed to the improvement of our manuscript. In the following section, you will find our detailed responses addressing each of your comments.

#### Specific Comments

##### Major Comments

*1. There does not appear to be a Table 2, but it is referenced in the text (page 7).*

Response: It seems there may have been some confusion regarding Table 2, which is referenced in the text. I’d like to clarify that Table 2 is indeed included in the manuscript.

*2. In the Discussion section on page 15, a reference is made to effect sizes in four outcome areas, yet no effect sizes were reviewed in the article.*

Response: Thank you for bringing this to our attention; we have corrected the mistake in the updated manuscript.

*3. Also in the Discussion on page 15, the word “power” is used: “The increased number of studies provided greater power in assessing variance heterogeneity and potential group differences.” Unless a specific power analysis was performed (if so, it should be discussed), the word “power” could be changed to “support” to reflect a review rather than an analysis.*

Response: Thank you for your suggestion. We have replaced the word “power” with “support” as per your suggestion to better align with the sentence.

*Similarly, on page 16, the term “meta-analysis” is used. Unless a secondary analysis of pooled data was performed, the term “meta-analysis” should be changed to “analysis.” If a secondary pooled analysis was performed, that should be defined and described in the body of the paper.*

Response: Thank you for your observation, and I apologize for any confusion. I want to clarify that a secondary pooled analysis was indeed conducted as part of the study, and it has been appropriately defined and described in the body of the paper.

*4. Page 16, Society for Healthcare Epidemiology of America is noted as an organization of interest for animal-assisted therapy, as is Pet Partners. The authors may want to consider including the global organization called International Association of Human-Animal Interaction.*

Response: Thank you for your insightful suggestion. We included the International Association of Human-Animal Interaction in the updated manuscript.

*5. On page 17, the authors cite lack of blinding as a limitation and introduction of bias. I would be curious to know how the authors propose blinding in studies that involve interactions with animals. I strongly suggest this sentence be removed.*

Response: Thank you for your suggestion. We have removed the sentence citing the lack of blinding as a limitation and the potential introduction of bias from the updated manuscript

*6. The work done by Hinic and others [* *3* *] was not a randomized controlled study as noted in Table 1. Please double-check that all studies listed are correctly labeled as randomized studies.*

Response: Thank you for bringing this to our attention. We have reviewed the studies listed in Table 1 and have corrected the labeling of the work by Hinic and others [3] to accurately reflect that it was not a randomized controlled study.

##### Minor Comments

*7. Check punctuation for spacing.*

Response: Thank you for your suggestion. We have reviewed and corrected punctuation for spacing in the updated manuscript.

*8. Check all abbreviations and use abbreviations after first use is defined.*

Response: Thank you for your guidance. We have carefully reviewed all abbreviations in the manuscript and ensured that they are defined upon their first use.

*9. Check capitalizations in midsentence (page 4: “Dog”; page 7: “Unrepresentative”).*

Response: We appreciate your attention to detail. Capitalization issues have been addressed and corrected in the updated manuscript.

*10. Page 6: “The articles should to be published in English.” Wrong tense—change to “were.”*

Response: Thank you for pointing out the tense issue. We have made the necessary correction in the updated manuscript.

*11. Page 1: Three categories of interventions were provided in section 2.4. It would strengthen the paper to include definitions of these categories for the reader.*

Response: Thank you for your constructive feedback. We have incorporated definitions for the three categories of interventions mentioned.

### Anonymous [4]

*Dear Authors,*

*First of all, your work’s topic is up-to-date and meticulously prepared. However, I still have a few questions/suggestions:*

Response: We sincerely appreciate and extend our gratitude for your valuable and relevant comments. Your input has been incredibly helpful in enhancing the quality of our manuscript. Below, you will find our point-by-point responses addressing each of your comments.

*1. In the Identification section, the total number of articles obtained from each database is given. It is recommended to give separate numbers for each.*

Response: Thank you for your suggestion. We have provided the separate number for each database in the updated manuscript.

*2. In the box below, the numbers are given as a total, but it may be more appropriate to give separate data for each item.*

Response: Thank you for your feedback. We have revised the presentation in the box below to provide separate data for each item.

*3. Can keywords be schematized in accordance with PICOS (Population, Intervention, Comparison, Outcomes, Study Design) in the literature review section?*

*Table...: Keywords used while browsing*

*Population*

*Intervention*

*Comparison*

*Outcomes*

*Study design*

Response: Thank you for your suggestion. We have now organized the keywords in accordance with PICOS in the literature review section.

*4. Has the quality of evidence been evaluated? If so, how was it done? This process can be explained by creating such a subtitle.*

- *How did you reduce the risk of bias in studies? Were the articles evaluated and scored separately among authors? Have these scores been analyzed?*
- *By whom and how was the screening done? I think that the most important limitation of this study is that it was scanned by a single person.*

Response: Thank you for your valuable input. We have addressed your concerns and included information on how the quality of evidence was evaluated, the process used to reduce the risk of bias in studies, and how the screening was conducted in the updated manuscript.

*5. In the section where general information is given for the last 16 articles, can it be added which disciplines are studied in particular? Since this subject is studied by various job groups, adding this information can enrich the data. If the mentioned situations are arranged, your article will contribute more to the literature.*

Response: Thank you for your suggestion. We have added information about the specific disciplines studied in the last 16 articles in the relevant section.

*6. What has been studied in previous systematic reviews? What are the original aspects of this work?*

*I include below some systematic review studies that may be relevant to the subject:*

- *Stern C, Lizarondo L, Carrier J, et al. The experiences and effectiveness of canine-assisted interventions (CAIs) on the health and well-being of older people residing in long-term care: a mixed methods systematic review protocol. PROSPERO. 2020. URL:* *https://www.crd.york.ac.uk/prospero/display_record.php?ID=CRD42020161235*
- *Whear R, McGill P, Orr N, et al. What are the effects of ‘robopets’ on the health and wellbeing of older people resident in care homes? A systematic review of qualitative and quantitative evidence. PROSPERO. 2017. URL:* *https://www.crd.york.ac.uk/prospero/display_record.php?ID=CRD42017081794*
- *Nabi N, McAloney-Kocaman K, Fleming M, Bain S. A systematic review exploring the effectiveness of animal-assisted therapy in improving the psychological well-being of incarcerated individuals. PROSPERO. 2022. URL:* *https://www.crd.york.ac.uk/prospero/display_record.php?ID=CRD42022314341*

Response: Thank you for your valuable input. We have thought from this aspect too and included the relevant work in the manuscript.

*If the mentioned situations are arranged, your article will contribute more to the literature.*

*I wish you good luck in your work.*

## Round 2 Review

Response: Thank you for your thoughtful comments and valuable insights regarding our meta-analysis. We appreciate your attention to detail and the opportunity to address the concerns you raised. We have updated the PICOS statement in the updated manuscript.

The following is our rationale for the specific keywords used in our search strategy and clarification of how they relate to the overall objectives of our study:

1. “Pain OR anxiety OR depression OR blood pressure OR BP OR heart rate OR HR”: Our focus on these specific health outcomes stems from the recognition that animal-assisted interventions (AAIs) have shown potential effects on psychological well-being (anxiety, depression) as well as physiological parameters (blood pressure, heart rate). Interactions with animals have shown potential in reducing blood pressure and heart rate, which are key indicators of anxiety and stress levels.
2. “Work-related stress OR workplace health OR employee well-being OR burnout” are critical factors that can significantly impact an individual’s overall health. Understanding how AAIs influence these aspects helps to recognize the influence of workplace factors on overall health, aligning with a holistic approach to health outcomes assessment.
3. “Tumor OR malignant OR carcinoma OR oncology OR hospitalization OR hospitalized patients OR inpatients” was searched to explore the potential applications of AAIs in health care settings, considering the well-documented positive effects of animal-assisted therapy on patients undergoing medical treatments, including those with cancer. While this may seem at odds with the workplace concepts, our intention was to provide a comprehensive overview of AAIs across various contexts, recognizing their multifaceted applications.

The following are some references pertaining to our search approach linked to the PICOS framework [5-7].

## Abbreviations

AAI: animal-assisted intervention
PICOS: Population, Intervention, Comparison, Outcomes, Study Design
